# Differential responses to acute administration of a new 5-HT7-R agonist as a function of adolescent pre-treatment: phMRI and immuno-histochemical study

**DOI:** 10.3389/fnbeh.2014.00427

**Published:** 2014-12-16

**Authors:** Luisa Altabella, Marco Sbriccoli, Francesca Zoratto, Anna Poleggi, Ramona Vinci, Enza Lacivita, Marcello Leopoldo, Giovanni Laviola, Franco Cardone, Rossella Canese, Walter Adriani

**Affiliations:** ^1^Department of Cell Biology and Neurosciences, Istituto Superiore di SanitàRome, Italy; ^2^Bambino Gesù Children's Hospital IRCCSRome, Italy; ^3^Dipartimento di Farmacia-Scienze del Farmaco, Università degli Studi di Bari “A. Moro”Bari, Italy

**Keywords:** hippocampus, nucleus accumbens, septum, limbic/cortical loop, LP-211, Ph-MRI

## Abstract

LP-211 is a new, selective agonist of serotonin (5-hydroxytryptamine, 5-HT) receptor 7 (5-HT7-R), which is part of a neuro-transmission system with a proposed role in neural plasticity and in mood, cognitive and sleep regulation. Adolescent subchronic LP-211 treatment produces some persisting changes in rats' forebrain structural and functional parameters. Here, using pharmacological MRI (phMRI), we investigated the effect of acute administration with LP-211 (10 mg/kg i.p.), or vehicle, to adult rats previously exposed to the same drug (0.25 mg/kg/day for 5 days), or vehicle, during adolescence (44–48 post-natal days); histology and immuno-histochemistry were performed *ex vivo* to evaluate neuro-anatomical and physiological long-term adaptation to pharmacological pre-treatment. The phMRI signal reveals forebrain areas (i.e., hippocampus, orbital prefrontal cortex), activated in response to LP-211 challenge independently of adolescent pre-treatment. In septum and nucleus accumbens, sensitized activation was found in adolescent pre-treated rats but not in vehicle-exposed controls. Immuno-histochemical analyses showed marked differences in septum as long-term consequence of the adolescent pre-treatment: increased level of 5-HT7-R, increased number of 5-HT7-R positive cells, and enhanced astrocyte activation. For nucleus accumbens, immuno-histochemical analyses did not reveal any difference between adolescent pre-treated rats and vehicle-exposed controls. In conclusion, subchronic LP-211 administration during adolescence is able to induce persistent physiological changes in the septal 5-HT7-R expression and astrocyte response that can still be observed in adulthood. Data shed new insights into roles of 5-HT7-R for normal and pathologic behavioral regulations.

## Introduction

The serotonin (5-hydroxytryptamine, 5-HT) receptor 7 (5-HT7-R), the most recently identified member in the family of G-protein-coupled serotonin receptors, is characterized by a widespread expression in the central nervous system, in the peripheral nervous system and in the periphery (Ullmer et al., 1995; Pierce et al., 1997; Nilsson et al., 1999; Neumaiera et al., 2001; Meuser et al., 2002). The topography of 5-HT7-R distribution in the brain, studied by several authors through the use of different techniques (Mullins et al., 1999; Neumaiera et al., 2001; Shirayama et al., 2001; Bonaventure et al., 2004), demonstrated predominant expression within the hypothalamus, thalamus, hippocampus, cortex, amygdala, and striatum. In line with this ample expression, 5-HT7-R has been linked to several functional roles, such as thermoregulation, learning and memory, hippocampal signaling, circadian rhythm, and sleep (Duncan et al., 1999; Hagan et al., 2000; Ehlen et al., 2001). The receptor is also involved in cognitive and emotional processes, including anxiety and depression (Hedlund and Sutcliffe, 2004; Meneses, 2004; Wesolowska et al., 2006; Mnie-Filali et al., 2011), and it has been recently proposed for a role in neurogenesis, synaptogenesis and dendritic-spine formation, especially during development (Kobe et al., 2012; Rojas et al., 2014).

This complex spectrum of functions attracted a great interest for the development of chemicals able to modulate the activity of this receptor in a targeted fashion. In recent years, different selective 5-HT7-R antagonist and agonist compounds have been developed. Among the latter, LP-211 is a novel agonist whose *in-vitro* pharmacological properties make it suitable for possible psychoactive effects (Leopoldo et al., 2008, 2011; Hedlund et al., 2010). Preclinical evidence in mice demonstrates consistent acute/sub-chronic effects onto exploratory motivation, anxiety-related profiles, and spontaneous circadian rhythm (Adriani et al., 2012; Romano et al., 2014). Moreover, given the putative role of 5-HT7-R in neural plasticity during development, we recently postulated that receptor stimulation may result in neuro-plastic changes leading to a persistent alteration on forebrain circuits. Such developmental effects of LP-211, originated by adolescent subchronic exposure, have been recently demonstrated in rats using either a behavioral approach (Ruocco et al., 2014a,b) or several different *in-vivo* magnetic resonance (MR) techniques (Canese et al., 2014).

The pharmacological MRI (phMRI), an application of functional MRI (fMRI), has started to represent a robust approach for the direct observation of drug action within the central nervous system (Gozzi et al., 2008; Kocsis et al., 2013), and—in the last years—it has become increasingly popular due to its non-invasiveness and relative low cost. The signal measured by either fMRI or phMRI is determined by local changes in the ratio of oxygenated to deoxygenated hemoglobin, and it is known as Blood Oxygenation Level Dependent (BOLD) signal. In response to neuronal activity, the locally elevated oxygen consumption leads to a feedback increase of blood flow, resulting in a rise of blood-oxygenation levels. In phMRI, changes in the BOLD signal are observed in response to a pharmacological stimulus. A previous study (Canese et al., 2011) revealed different phMRI responses to selective or non-selective blockade of serotonergic pathways, suggesting that the use of selective compounds, and noteworthy agonists such as LP-211, could give a deeper insight into the serotonin system.

In this work, we investigated the differential response to acute LP-211 administration in rats, as a function of adolescent subchronic exposure to the same drug. The use of phMRI allowed us a deeper view into persistent, long-term changes triggered by developmental 5-HT7-R stimulation, by investigating the carry-over sensitization of acute, hemodynamic effects induced by LP-211. Only those brain areas, displaying a differential drug-evoked phMRI signal as a function of pre-treatment, were then selected for *ex vivo* histological and immuno-histochemical analyses, in order to evaluate underlying neuro-anatomical and neuro-physiological changes.

## Materials and methods

All experimental procedures were approved by the Institutional Animal Survey Board on behalf of the Italian Ministry of Health (formal license to G.L.). Procedures were in close agreement with the European Communities Council Directive (86/609/EEC) and Italian law. All efforts were made to minimize animal suffering, to reduce the number of animals used, and to utilize alternatives to *in-vivo* techniques, if available.

### Subjects

Twenty male Wistar rats from our colony were housed in pairs inside polycarbonate cages (42.5 × 26.6 × 18.5 cm) with sawdust bedding, in an air-conditioned room (21 ± 1°C, relative humidity 60 ± 10%), on a 12-h reversed light-dark cycle (lights off at 7.00 a.m.). Food (Altromin-R, A. Rieper S.p.A., Vandoies, Italy) and tap water were provided *ad libitum*.

### Pharmacological treatment

Rats were randomly assigned to receive a sub-chronic intraperitoneal (i.p.) administration of LP-211 (LP, 0.250 mg/kg/day, *n* = 10), or vehicle (VEH, 1% DMSO in saline, *n* = 10) for 5 days during the adolescent phase (43–47 post natal days, PND). The drug dosage was chosen according to previous data obtained in mice and rats (Adriani et al., 2012; Canese et al., 2014; Romano et al., 2014; Ruocco et al., 2014a,b).

At adulthood (63–75 PND; weight range: 340–430 g), rats underwent phMRI, with an acute challenge of either LP-211 (10 mg/kg i.p.), or vehicle. Our aim was to measure acute response to LP-211 challenge, modulated by the different pre-treatments. Animals were therefore subdivided into three final experimental groups, according to the substance administrated in the adolescent phase (subchronic pre-treatment) and during phMRI analyses (acute administration): 1. the adolescent subchronic VEH + acute VEH (VEH+VEH) and the adolescent subchronic LP-211 + acute VEH (LP+VEH) were collapsed, generating a single “vehicle-challenge” group (VEH), since no differences between these two groups were observed in preliminary phMRI data analyses; 2. adolescent subchronic VEH + acute LP-211 (VEH+LP: acute challenge in drug-naive rats); 3. adolescent subchronic LP-211 + acute LP-211 (LP+LP: sensitized response to the challenge).

### Preparation of animals for phMRI

Anesthesia was induced with isoflurane (Esteve veterinaria, Spain, 5% for induction and 2.5% during the set-up) in O_2_ and then rats were intubated and mechanically ventilated [90 bpm, volume depends on animal weight following the formula: strokes volume (ml) = animal weight (g) × 0.0062]. Artificial ventilation allows triggering all functional acquisitions at the same instant of breath cycle, thus minimizing motion artifacts and preventing drug-induced alterations in the respiratory rate when the LP-211 challenge is injected.

Rats were fixed on the cradle using a stereotaxic head frame to reduce head movements. A cannula was inserted subcutaneously to administrate a bolus of medetomidine (Domitor, Pfizer Germany, 0.05 mg/kg in 0.5 ml) and then connected to the infusion line for continuous medetomidine administration (0.1 mg/kg/h in 1 ml/h). The continuous infusion of the anesthetic started 15 min after bolus; at the same time, isoflurane was reduced to 0.6%. A second cannula was inserted into the peritoneum and connected to an infusion line for remote drug administration during the MRI session. An integrated heating system allowed maintaining the animal body temperature at 37.0 ± 0.1°C.

Rats were monitored during MR scanning using a MRI-compatible pulse oximeter (MouseOx, Starr Life Sciences Corp) that provides on-line measures of heart rate, oxygen saturation, and pulse distension (a surrogate parameter for blood pressure; its measured values depend on the intensity of the pulse-oximeter signal, therefore we normalized its actual value to a percentage of maximum in the time course of each animal). After the scan, medetomidine was antagonized using the antidote, atipamezole (Antisedan, Pfizer Germany, double dose with respect to medetomidine in 0.5 ml s.c.) that ensures a fast awakening and recovery of all animals. A wash out period of at least 1 month was left before sacrifice.

### phMRI protocol and acquisition

Experiments were performed on a VARIAN/Agilent Inova MRI/MRS system operating at 4.7 T equipped with an actively shielded gradient system (max 120 mT/m, 11 cm bore size). A volume coil of 6-cm diameter was used for transmission in combination with an electronically decupled receiver-only surface coil (Rapid Biomedical, Rimpar, Germany). The shape of this receiver coil (3 cm long, 3 cm wide, and 1 cm high) was designed to optimally fit the dorsal surface of the rats' heads centered over the forebrain regions.

**Figure 1:**
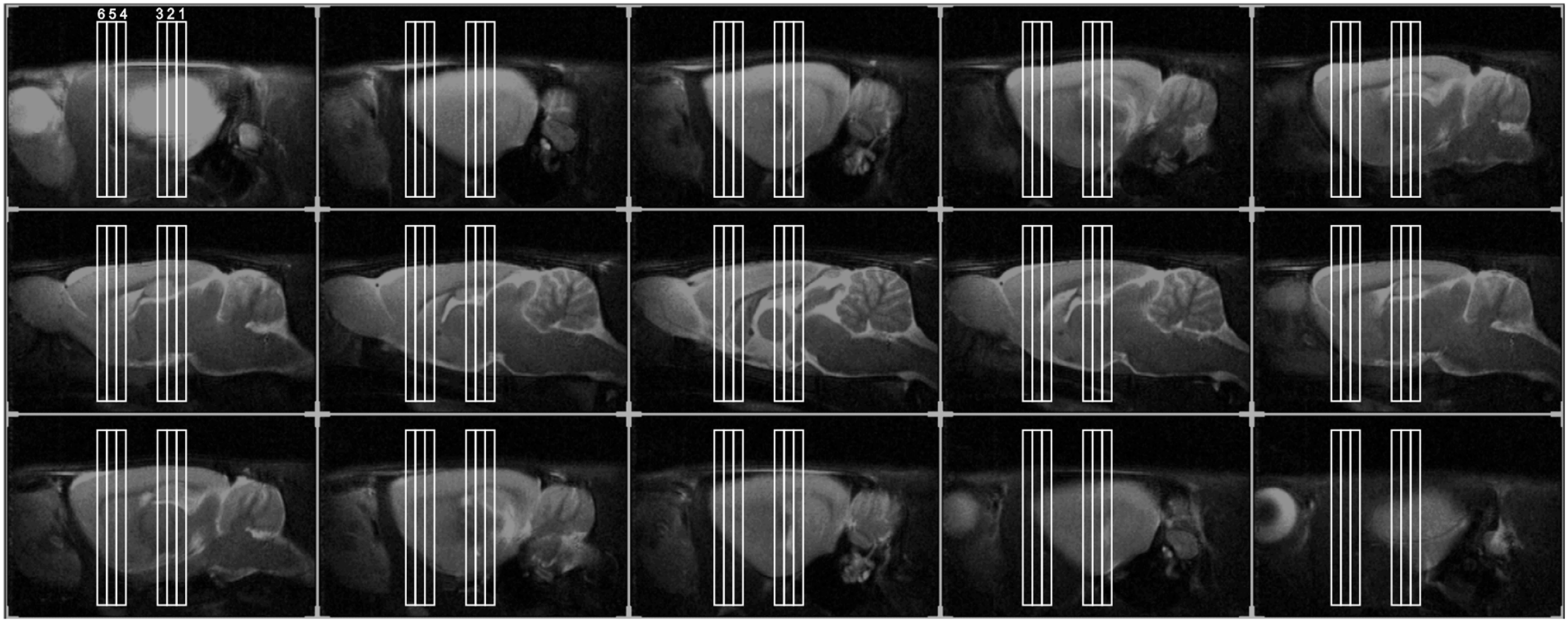
**Positioning of the six slices for phMRI, overlaid on the anatomical sagittal fast spin-echo images taken from an individual representative rat**. We acquired six slices in total: three consecutive slices (thickness 1 mm), centered on the hippocampus (Hip, slice 1, 2, 3), as well, after a gap of 3 mm, other three slices. These were centered, respectively, on: dorsal striatum (dStr, slice 4), on the fibers between the prefrontal cortex and striata (slice 5), on prefrontal cortex (PFC, slice 6).

Gradient echo scout images were used to detect position of each animal's head inside the magnet. Then, fast spin-echo sagittal anatomical images (TR/TEeff = 3000/15 ms, 15 consecutive slices of 1 mm thickness, FOV = 40 × 40 mm^2^, matrix of 256 × 256, 4 averages, voxel resolution = 0.31 × 0.31 × 1 mm) were used to accurately position axial images for the pharmacological study. In order to have a reproducible positioning of acquired slices, we considered the forceps minor of the corpus callosum as reference (Canese et al., 2009) as shown in Figure 1.

Echo planar breath-triggered images were acquired (*TR* ≈ 4000 ms, *TE* = 23 ms, matrix 64 × 64, FOV 25 × 25 mm^2^, slices: 6, thickness 1 mm, 1 average). After consecutive image collection for about 12 min (20 baseline images), rats received LP-211 (10 mg/kg i.p.) or vehicle (1 ml/200 g body weight). Images were then collected for further 24 min (40 post-challenge images).

### phMRI data and statistical analyses

Data were analyzed by a home-made program (developed in Matlab, Mathworks Inc.) as previously described (Canese et al., 2009, 2011). During the pre-processing, images were realigned in order to reduce the artifacts due to animal movements during data acquisition (Canese et al., 2009). Once the images were realigned, they were then restored in order to increase the signal-to-noise ratio (SNR). In order to efficiently smooth the rough data, we implemented a procedure based on a moving average data interpolation. Then, two-step statistical approaches were conducted in parallel (Canese et al., 2011) on the acquired datasets.

#### First step

We constructed a template for each group: after selecting the dataset of one subject as a reference for the group, the datasets of the other subjects were transformed by means of a poly-nomial transformation (Goshtasby, 1988). Once the image sequences had been co-registered to the reference images, the software averaged the whole data acquired from all individual datasets of each group in order to obtain one template per group. Within each template, significant temporal alterations in BOLD signal profiles were assessed pixel-by-pixel, through a random effect analysis comparing a post challenge time window (five-point wide window centered on the signal maximum) to the mean baseline signal, with a two tailed Student *t*-test (*p* < 0.065, Bonferroni corrected).

#### Second step

**Figure 2:**
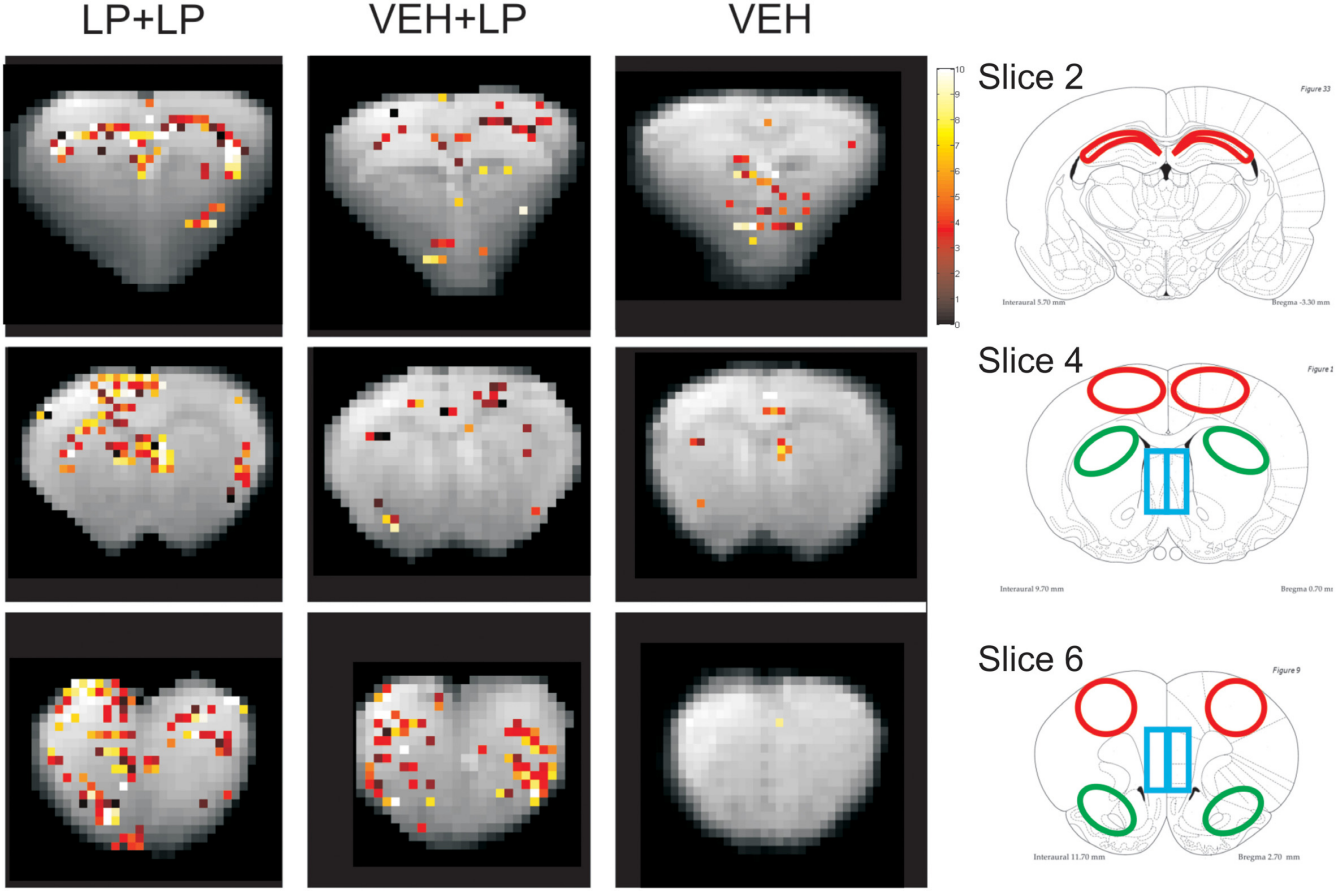
**Right panels: localization of ROIs**. **Left panels:** BOLD activation maps (the Student-*t*-values, in color scale), overlaid on anatomical images (in gray scale). Both are taken from the three brain templates, originating from rats receiving: vehicle challenge (VEH), LP-211 acutely only (in drug-naive subjects) i.e., following adolescent vehicle exposure (VEH+LP), LP-211 also in acute but following adolescent subchronic pre-treatment (LP+LP). Only pixels with significant values (*p* < 0.065 for slice on Hip and *p* < 0.05 for the others) are illustrated. ROIs were taken with the help of the Atlas (Paxinos and Watson, 1998) from: the CA1-CA2 fields of Hip (red regions at −3.30 from bregma, slice 2); from the dSTR, septum and frontal cortex (see green, blue, red ROIs, respectively, at +0.7 from bregma, slice 4); from the nucleus accumbens, medial prefrontal cortex, dorsal prefrontal cortex (see green, blue, red, ROIs, respectively, at +2.7 from bregma, slice 6).

We extracted BOLD signal intensity timecourses, i.e., one curve for each individual animal, from several brain areas. These regions-of-interests (ROIs) were selected (manually, on the template images, Figure 2) based on those areas showing apparent activation in LP+LP but not VEH+LP templates. We therefore selected: hippocampus (Hip) in slice 2; septum, dorsal striatum (Str) and frontal cortex in slice 4; medial prefrontal cortex, dorso-lateral prefrontal cortex and nucleus accumbens (NAcc) in slice 6, according to the activation maps of group templates.

In the timecourses for the extracted BOLD signal curves, each temporal point was obtained by averaging the BOLD signal intensity values across all chosen-ROI pixels at that time point. In such BOLD timecourses, each time point was expressed as the percentage change of the signal intensity from the first time point. These BOLD timecourses underwent normalization of baseline and de-trending. In particular, the baseline mean values (estimated as an average of the first 20 time points) were subtracted from each BOLD timecourse. This, in order to decrease inter-individual variability due to random (instrumental and biological) fluctuations in the first timepoint. Then, for each individual rat, a linear regression fit was applied to the first 20 and last 15 time points, in order to estimate the temporal drift of the curve. This drift line was then subtracted from the entire BOLD timecourse (Canese et al., 2009, 2011).

Like for group templates, animals receiving acute vehicle challenge (LP+VEH and VEH+VEH) where merged in a single group (VEH): this, since preliminary analyses yielded no differences between these two groups, as expected. Split-plot ANOVA was thus performed, with group (three levels: LP+LP vs VEH+LP vs VEH) as between subjects factor, time (40 points after injection, 24 min) and side (right vs left hemispheres except for septum and medial prefrontal cortex) as within subject factors. Analyses were performed on the post-challenge curves, in search for drug-induced effects. Tukey's Honestly Significant Difference (HSD) test was used for multiple *post-hoc* comparisons (Abdi and Williams, 2010).

### Tissue sampling and processing

At sacrifice, brain hemispheres were fixed in 4% buffered formaldehyde and blind coded brain slices were obtained from four standard coronal sections (2-mm thick). The slices were washed in running water, dehydrated in graded alcohols, cleared in xylene and finally embedded in paraffin wax. 5-μm-thick sections were cut on microtome, and collected onto glass slides for histological and immuno-histochemical analyses.

#### Histology and immuno-histochemistry

For histological analyses, the slides were deparaffinized, rehydrated, and stained with haematoxylin/eosin or with luxol fast blue/eosin for the precise identification of the brain structures. For immuno-histochemistry, sections were collected on Super Frost Plus slides and immunostained according to the ABC method for brightfield examination.

Briefly, sections were deparaffinized, rehydrated and subjected to antigen retrieval by microwaving in citrate buffer. The slides were then treated with 3% hydrogen peroxide in methanol to block endogenous peroxidases activity, rinsed in PBS, blocked for 1 h in PBS containing 3% normal goat serum and then incubated overnight at 4°C with one of the following antibodies: 5-HT7-R (cod. NBP1-46598, Novus Biologicals, rabbit, 1:1000 dilution); post synaptic marker PSD95 (cod. NBP1-47642, Novus Biologicals, mouse, 1:500 dilution); neuro-filament NF-L (DA2) (cod. NB 300-132, Novus Biologicals, mouse, 1:250 dilution); GFAP for astrocytes (cod. M0761, DAKO, mouse, 1:100 dilution).

Subsequent antibody detection involved incubation with the appropriate biotinylated secondary antibody for 1 h (1:200 dilution, Vector Laboratories, Italy) at room temperature, followed by incubation with the avidin-biotin-peroxidase complex (Vectastain ABC-Elite kit, Vector Laboratories, Italy) according to the manufacturer's instructions. The samples were stained with 3′-3′diaminobenzidine (DAB, Sigma, Italy) as chromogen to visualize the reaction product and then lightly counterstained with haematoxylin.

For immuno-fluorescence, sections were processed as above but the antibody detection was performed through 1 h of incubation with Alexa Fluor 488 secondary antibody (1:300 dilution, Invitrogen, Italy). In order to obtain comparable data and a more consistent staining, each antibody was tested on both groups in the same immuno-histochemical run.

For the count of 5-HT7-R and GFAP positive cells and for densitometric measures, anatomical landmarks were used as optical reference so that equal areas at the rostrocaudal levels were analyzed for each animal. Three non-consecutive serial sections from each brain were photographed under 20× magnification, with constant light conditions, and the labeled cells were counted manually in selected brain areas (i.e., nucleus accumbens and septum). Staining intensities were quantified on immunofluorescent slides using ImageJ software (NIH, http://rsb.info.nih.gov/ij). 5-HT7-R intensities were calculated using the mean intensity value of the 5-HT7-R positive cells. For that, RGB images were splitted, the green channel was selected and 8-bit converted. Images were then thresholded and ROIs selected for quantification. Optical densities of the ROIs were recorded and expressed as values on a gray scale, ranging from 0 (black) to 255 (white). For statistical analysis, student *t*-test was used.

## Results

Heart rate and pulse distension, measured pre and post challenge during the phMRI study, are summarized in Table 1. Typical time courses are shown in Supplementary Figure 1. No statistical differences were found in the data between the LP-211 challenge and vehicle controls [ANOVA with repeated measurements, *F*_(1, 8)_ = 2.71, n.s., *F*_(1, 8)_ = 0.03, n.s., respectively], confirming that LP-211 challenge did not alter the depth of anesthesia in rats. Oxygen saturation is not relevant in these experiments because animals were mechanically ventilated with oxygen at 99% (instead of air) during MR measurements. Therefore, this parameter was constant during the whole experiment.

**Table 1:** **Physiological parameters in rats during Ph-MRI measurements, before (Pre) or after (Post) the challenge administration**.

	**Heart rate (bpm)**		**Pulse distension (%)**	
**Experimental group**	**Pre**	**Post**	**Pre**	**Post**
(VEH or LP)+LP	234.36 ± 36.20	218.20 ± 32.61	89 ± 4%	73 ± 9%
VEH	226.70 ± 23.53	219.74 ± 20.58	84 ± 2%	66 ± 10%

A significant reduction in pulse distension between pre and post challenge is present in all groups [time, *F*_(1, 8)_ = 41.45, *p* < 0.001]. This reduction cannot be attributed to LP-211 challenge or pre-treatment since it is present also in vehicle controls, and it is probably due to the prolonged anesthesia or to cardiovascular readjustment to the volume of injection. Finally, time courses of physiological parameters measured during MR scan pre and post challenge were compared to time courses of BOLD curves and we found they were temporally uncoupled.

Our findings are not in conflict with the increased wakefullness in rats due to LP-211, as reported in literature (Romano et al., 2014). In fact, in our case, rats were anesthetized with a double anesthetic (isoflurane and medetomidine) that likely overcomes the behavioral effect of LP-211 already reported.

### Templates' activation maps, bold timecourses

No consistent activation was found in the VEH template. As for drug challenged groups, activation maps showed BOLD signal hyper-intensity within several regions, with different extension and intensity depending on the adolescent, subchronic pre-treatment. Areas of a positive BOLD effect (i.e., regions whose pixels exceeded the threshold of significance) were detected, within the LP+LP but not the VEH+LP group, for the hippocampus (CA1-CA2 fields, slice 2), septum, frontal cortex, and part of dorsal striatum (slice 4), as well as for medial prefrontal cortex, dorso-lateral prefrontal cortex and the nucleus accumbens (slice 6). The orbital prefrontal cortex was the only region showing clear-cut activation in templates for both VEH+LP and LP+LP groups.

Therefore, as shown in Figure 2, the VEH+LP group showed a differential response to the challenge with respect to the LP+LP group. In particular, for drug-naive animals receiving LP-211 for the first time as an acute challenge (i.e., VEH+LP template), non-localized and/or widespread effects were observed, with just scattered pixels or reduced extension of activation, in hippocampus and at the level of the nucleus accumbens. In this template, the septum, frontal cortex and dorsal striatum, as well as dorso-lateral and medial prefrontal cortex, did not show any significant response; therefore, for the latter areas, the VEH+LP and VEH templates were undistinguishable, suggesting no effect at all of an acute LP-211 challenge in drug-naive rats.

**Figure 3:**
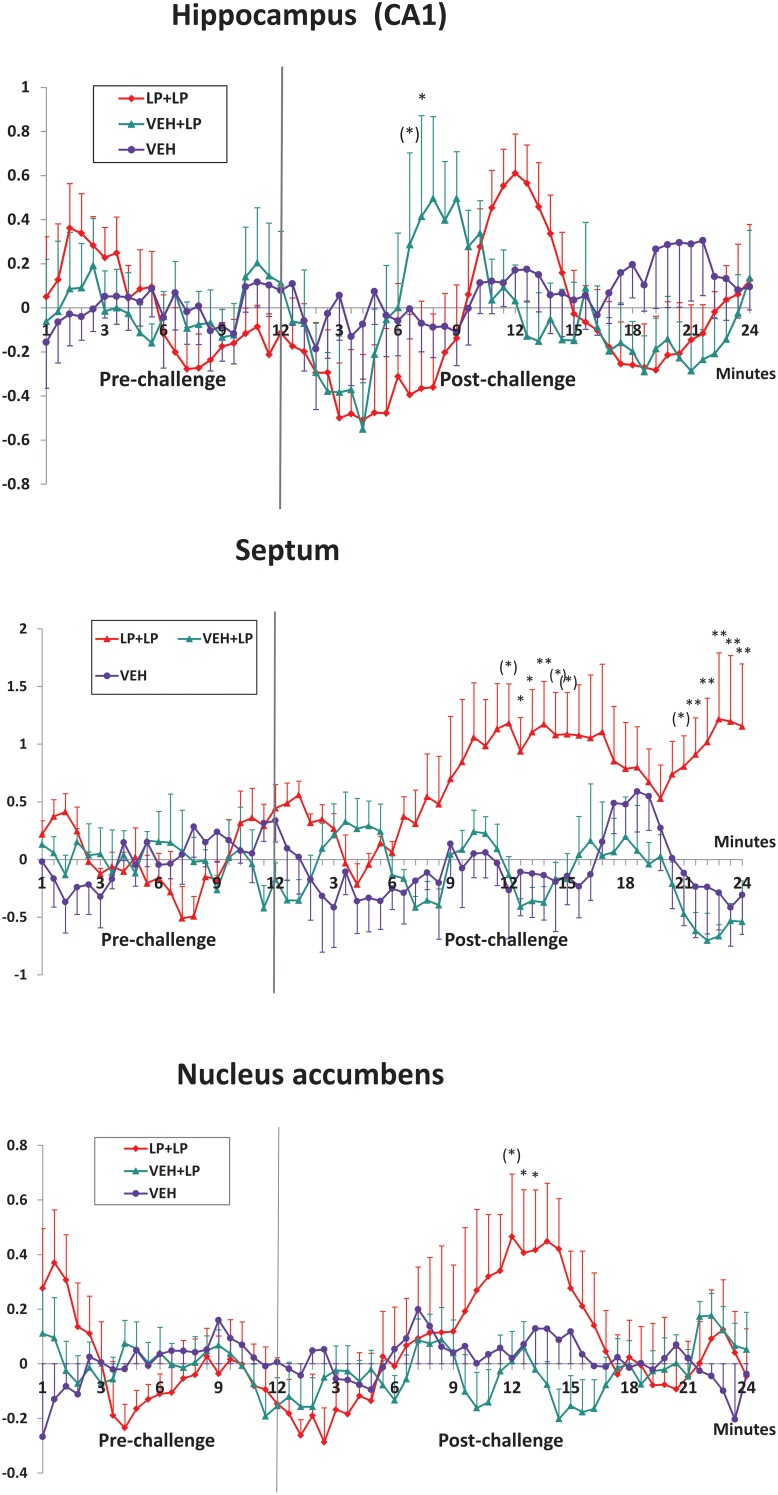
**Mean ± s.e.m. change (%) in BOLD signal timecourses, in the CA1-CA2 fields of Hip, septum and nucleus accumbens (ROIs shown in Figure 2), for rats receiving vehicle challenge (VEH), LP-211 acutely only (in drug-naive subjects) i.e., following adolescent vehicle exposure (VEH+LP), LP-211 also in acute but following adolescent subchronic pre-treatment (LP+LP)**. Time after the challenge is expressed over ten 3-min intervals, obtained by averaging the originally acquired data in ten 5-point-wide time-windows. Significant *post-hoc* Tukey comparison (LP+LP vs VEH+LP groups) are indicated as follows: (^*^)0.05 < *p* < 0.1; ^*^p < 0.05; ^*^p < 0.01.

Localization of the seven regions, for time-courses extraction and analysis, is shown in Figure 2 right panel. Analyses of the BOLD timecourses, individually extracted from subjects, confirmed a differential activation for just three out of the seven chosen areas (see Figure 3): for hippocampus, nucleus accumbens, and septum. Data showed that the BOLD effects differed between LP+LP and VEH+LP experimental groups. Significant interactions time × treatment were indeed found only in septum [*F*_(80, 360)_ = 1.43, *p* = 0.015], in hippocampus [F_(80, 360)_ = 1.70, *p* = 0.006], and in nucleus accumbens [*F*_(80, 280)_ = 1.55, *p* = 0.005]. For both septum and nucleus accumbens, the *post-hocs* confirmed that a BOLD effect was evident in LP+LP and absent in VEH+LP curves. For the hippocampus, an activation signal was present in both the LP+LP and VEH+LP groups (though with different intensities and temporal outline, see Figure 3). This, together with the scattered points already mentioned by VEH+LP template inspection, suggested that this area was indeed activated in drug-naive rats, after a single acute LP-211 administration.

For medial and dorso-lateral prefrontal cortex, frontal cortex, as well as dorsal striatum (all of which appeared to be activated on LP+LP template but clearly devoid of activated pixels on VEH+LP template), ANOVAs did not confirm the profile (data not shown): as such, the pixels activated on template may well be a false positive. The ANOVA on timecourses was not necessary for orbital prefrontal cortex, as this area was clearly activated in both LP+LP and VEH+LP groups.

### *Ex vivo* histology and immuno-histochemistry

We performed histological examinations of luxol fast blue and haematoxylin-eosin stained slides obtained throughout the whole brain. This morphological analysis showed no relevant alteration in LP+VEH vs VEH+VEH animals. In-depth immuno-histochemical analyses, for the evaluation of neuro-anatomical and neuro-physiological changes, were then conducted in two selected brain areas. We chose the nucleus accumbens and the septum, the only two regions with positive phMRI activations for the LP+LP group only, confirmed both in template maps and in BOLD timecourse analyses (depicted in Figures 2, 3). These areas could possibly have developed stable neuro-anatomical or neuro-physiological changes, induced by subchronic treatment with LP-211 during adolescence.

The *ex vivo* investigation for prefrontal and frontal cortex regions as well as dorsal striatum was not performed, because these areas resulted not to significantly differ, as a function of pre-treatment, in the ANOVA analyses. Conversely, for the orbital prefrontal cortex and the hippocampus, activation signal was present, after acute LP-211 administration, in both the LP+LP and VEH+LP groups; therefore, an *ex vivo* analysis in these two regions was out of the purposes of present study.

**Figure 4:**
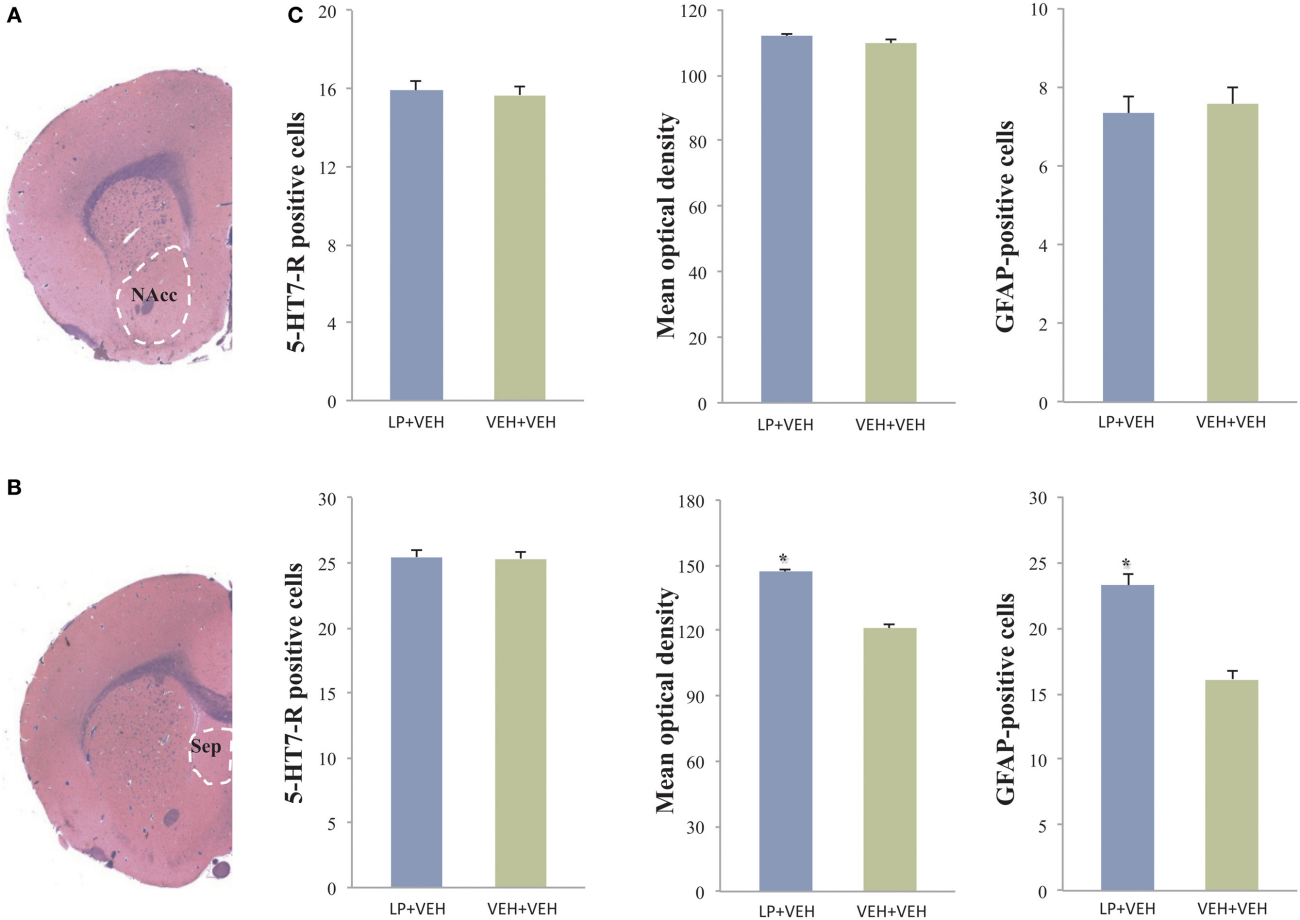
**Analysis of 5-HT7-R and GFAP immunoreactivity in the nucleus accumbens (NAcc) and in the septum: statistically significant differences were observed only in the septum**. Here, animals subchronically pre-treated during adolescence with LP-211 drug showed an increased 5HT7-R optical density and an increased number of GFAP-positive cells with respect to VEH-exposed control group **(C)**. The values are given as mean ± *SE*; asterisks denote a statistically significant difference (*p* < 0.05). No differences for 5HT7-R were observed by cell count in both areas. **(A,B)** are representative photomicrographs at the level of the NAcc and septum in Luxol fast blue-stained slides.

**Figure 5:**
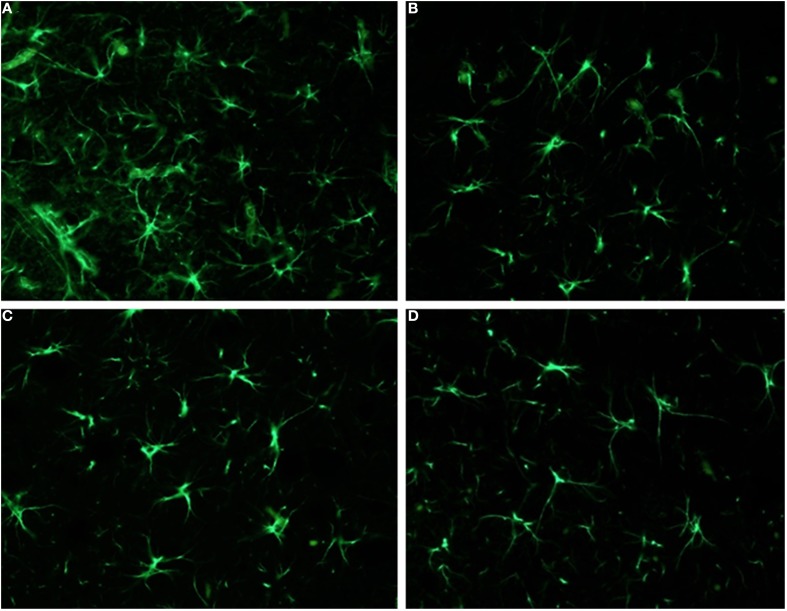
**Analysis of GFAP immunoreactivity in the nucleus accumbens (NAcc) and in the septum: significant differences were observed only in the septum**. Here, animals subcronically pre-treated during adolescence with LP-211 drug **(A)** revealed an increased GFAP immunoreactivity, with respect to the VEH-exposed control group **(B)**. No differences were conversely observed in the nucleus accumbens **(C,D)**. Original magnifications 40×.

Regarding the 5-HT7-R expression, counting of immunoreactive cells in the septum revealed no differences in the total number of 5-HT7-R expressing cells between the two groups. Conversely, the analysis of the 5-HT7-R optical density revealed a significant increase in immunoreactivity for the group subjected to adolescent pre-treatment, compared to adolescent VEH-exposed controls (Figure 4). These results indicate increased levels of receptors expressed by septal cells, as a long-term consequence of adolescent LP-211 treatment. Besides the 5-HT7-R changes, we found an increase in GFAP immunoreactivity (Figure 5), suggesting persistent rearrangement with an increased response of astroglial cells in adolescent-LP-211 pre-treated animals. No differences were observed with the post-synaptic and with the neuro-filament markers.

In the nucleus accumbens, neither the cell counts nor optical density analyses of 5-HT7-R positive cells revealed any difference between adolescent drug pre-treated animals and VEH-exposed controls (see Figure 4); likewise, no differences were observed with the other markers. Such results suggest that the subchronic pre-treatment with LP-211 during adolescence does not produce an immuno-histochemically detectable effect on this brain area, at least regarding the selected markers.

## Discussion

The present work was aimed to study the long-term sensitizing effects of the adolescent, subchronic administration of LP-211, a novel 5-HT7-R agonist. To do this, a two-level approach was adopted: first, adult rats underwent phMRI analyses (with an LP-211 challenge) following a previous adolescent exposure, to identify LP-211 sensitized areas which were not acutely responsive in drug-naive rats; once identified, these (two) areas were studied *ex vivo*, at the histological and immuno-histochemical level, to detect the presence of stable neuro-anatomical and biochemical changes.

General anesthesia is usually induced in rodents during phMRI studies in order to reduce motion artifacts, to minimize the stress produced by prolonged restraint and MRI gradient noise, and to facilitate animal handling (Lukasik and Gillies, 2003; Sicard et al., 2003; Steward et al., 2005). Unfortunately, general anesthesia may perturb cerebral blood flow and metabolism, as well as electro-physiological, cardio respiratory, and other physiological parameters; finally, it can suppress neuronal activity reducing the BOLD response (Gozzi et al., 2008). Recent studies reveal that BOLD response to the same challenge may change from negative to positive, or differ in amplitude, with different anesthetic regimes (Sommers et al., 2009; Hodkinson et al., 2012; Liu et al., 2012). Also, the pharmacological challenge in itself can produce cardio respiratory physiological responses that may alter hemodynamic measurements in brain. The particular sensitivity of phMRI to natural and anesthesia-induced fluctuations in vital parameters is a well-documented issue, affecting the quality and interpretation of phMRI results. For such reasons, in the last years several studies have tried experimental procedures to achieve the most reliable and reproducible phMRI acquisition protocol (Steward et al., 2005; Weber et al., 2006; Ferrari et al., 2012).

For these reasons, the present work exploited a protocol for sedation and anesthesia consisting of two anesthetic compounds (isoflurane and medetomidine), under continuous supply, associated with intubation, and mechanical ventilation, to assure stability in physiological parameters: such a protocol is suitable for prolonged investigations (Weber et al., 2006; Fukuda et al., 2013). This protocol also assures a fast recovery of the animals and therefore it is suitable for longitudinal studies as well as for further behavioral testing (Canese et al., 2014). Once optimized, this setting (anesthetic regimen combined to the respiratory trigger) allowed to acquire phMRI scans of the desired resolution, with signal alterations as low as 0.5% and strongly reduced motion artifacts.

Moreover, physiological parameters (heart rate and pulse distention) measured during MR scan, pre and post challenge, did not show differences due to LP-211 administration. We can exclude any bias due to cardiovascular rearrangements or systemic alterations, and therefore attributed to LP-211 any effect observed in BOLD signal variations.

In this way, we found robust and significant results by using and comparing data obtained with two different and parallel statistical approaches (Canese et al., 2011). By using this combined approach, we were able to identify significant alterations in several forebrain areas. The orbital prefrontal cortex and hippocampus were the only regions showing a clear-cut activation due to drug challenge, with no differences between adolescent-LP-211 pre-treated (LP+LP) and adolescent-VEH exposed (VEH+LP) groups; therefore, these were not further investigated *ex vivo*. Conversely, in the absence of acute response to LP-211 (10 mg/kg i.p.) within drug-naive subjects, two areas showed a sensitized response to the same drug due to previous developmental exposure. Indeed, in the septum as well as in the nucleus accumbens, BOLD response was evident within the LP+LP group but not in the VEH+LP one. A somewhat similar profile seemed to emerge for frontal and prefrontal cortex as well as (part of) dorsal striatum, at least in template activation maps, but this was not confirmed by ANOVA analyses on BOLD timecourses.

The suggestion of a persistent modification in neurocircuitry, supported by recent data (Canese et al., 2014), was further examined with immuno-histochemistry. In the absence of detectable changes for the nucleus accumbens, the septum revealed the most interesting differences between adolescent-LP-211 pre-treated vs. adolescent-VEH exposed rats after *ex vivo* immuno-histochemical examination. In particular, sub-chronic administration of LP-211 during adolescent phase exerted a direct and long-lasting effect in the septal area, with a specific increase of 5-HT7-R immunoreactivity coupled with an increased astrocytes response (as observed by GFAP immunoreactivity).

These two aspects may well be related. The observed increase in astrocytes response within the septum could represent a specific, yet secondary response to an increased 5-HT7-R immunoreactivity in local neurons, produced by the LP-211 adolescent pre-treatment. Together with this, it is possible that the increased 5-HT7-R immunoreactivity, observed in the septum, derives, at least in part, from an increased receptor expression in activated astrocytes. Consistently, 5-HT7 receptors have been classically reported in glial processes within the supra-chiasmatic nucleus (Glass and Chen, 1999; Belenky and Pickard, 2001), in astrocytes from the frontal cortex (Shimizu et al., 1996), and in primary astrocyte cultures (Hirst et al., 1997).

The idea of astrocytes as primary players in the pathophysiology of the CNS is not new: astrocytes are key elements that participate in all essential CNS functions. They represent a wide and heterogeneous population of cells whose activity is finely regulated on the basis of specific functions and responses of their relevant neuronal population (Fellin, 2009). In recent years, astrocytes are emerging as relevant and active elements in brain physiology, where they act by exchanging information with the synaptic neuronal elements, responding to synaptic activity and regulating synaptic transmission. The term “tripartite synapse” is now used to represent the bi-directional communication between astrocytes and neurons at the synapse's interface (Araque et al., 1999). In line with this, a major role of astrocytes is the uptake of neuro-transmitters, mainly glutamate and GABA, therefore contributing to modulate local network transmission. Variations in neuro-transmitter levels (mainly glutamate) were already observed, after adolescent subchronic LP-211 administration, in previous work by our group: through amino-acid detection *ex vivo*, within prefrontal and striatal areas (Ruocco et al., 2014a,b) and through MR spectroscopy, within the hippocampus (Canese et al., 2014). We could speculate that similar variations might also occur in the septum, and this would represent a further stimulus for an increased astrocytes immunoreactivity. We did not observe any variation in neuro-filament and synaptic markers, supporting the conclusion of a predominantly glial modification.

Surprisingly, we have not found immuno-histochemical variations in the nucelus accumbens, the other area where sensitized BOLD responses to LP-211 were observed with our phMRI analyses. Although undetectable in our hands, we cannot exclude that adolescent LP-211 administration resulted in variations of the same markers, which were below the detectable level with immuno-histochemistry but would become evident if tested with other techniques. In any case, a set of morphological and functional changes (in the dendritic tree and network connections) were already revealed for the nucleus accumbens in our previous work (Canese et al., 2014). It is therefore possible that, in the absence of changes on the selected markers, persistent modifications could subtend the activation of other proteins or effector patterns downstream of the 5-HT7-R activation, and/or the mirroring of drug-evoked effects originated from other areas then projecting into the nucleus accumbens, as opposed to the direct effect clearly observed in the septum. Accordingly, we found in our previous work (Canese et al., 2014) that long-term functional modifications, produced by adolescent LP-211, were tapping onto the limbic loop in general and nucleus accumbens in particular. If so, the altered phMRI signal could be interpreted as an indirect effect (of the adolescent LP-211 administration), primarily evoked in another node of the loop (e.g., the hippocampus or orbital prefrontal cortex, actually stimulated). Conversely, while the septum was persistently modified in its anatomy (present data), it did not emerge as part of the functional connectivity alterations induced by adolescent LP-211 in our previous data (Canese et al., 2014).

As a final remark about drug dosage, LP-211 (10 mg/kg) has been used in various *in vivo* studies (Hedlund et al., 2010; Martinez-Garcia et al., 2014; Meneses et al., 2014), and effects possibly mediated by the activation of 5-HT1A or 5-HT2B receptors were not observed (i.e., body temperature, cardiac failure, respectively). Therefore, although the dosage is relatively high, we can confidently assume that LP-211 is still selective to 5-HT7 receptor.

## Conclusion

In summary, our results suggests that subchronic LP-211 administration during the adolescent phase is able to induce physiological changes in the 5-HT7-R expression and in astrocytes response, within the septum, that can still be observed in adulthood. The increased number of GFAP-positive cells within the septum, found in the present work, is not yet related to the evidence of a rearranged connectivity, recently found within the whole limbic loop (Canese et al., 2014). Therefore, further studies are required to better clarify the nature and the output of such enduring changes.

### Conflict of interest statement

The authors declare that the research was conducted in the absence of any commercial or financial relationships that could be construed as a potential conflict of interest.

## Supplementary material

Supplementary Figure 1 **An example of physiological time courses in a LP-211 challenged rat**. After drug administration, there is only a transient increase of pulse distension and a slight and stable increase of heart rate. None of these time courses is coupled with the BOLD one, thus excluding any bias due to systemic effects.

## References

[B1] Abdi H. Williams L. J. (2010). Tukey's honestly significant difference (HSD) test. Encyclopedia Res. Des. 1, 1–5 10.4135/9781412961288.n181

[B2] Adriani W. Travaglini D. Lacivita E. Saso L. Leopoldo M. Laviola G. (2012). Modulatory effects of two novel agonists for serotonin receptor 7 on emotion, motivation and circadian rhythm profiles in mice. Neuropharmacology 62, 833–842. 10.1016/j.neuropharm.2011.09.012 21945717

[B3] Araque A. Parpura V. Sanzgiri R. P. Haydon P. G. (1999). Tripartite synapses: glia, the unacknowledged partner. Trends Neurosci. 22, 208–215. 10.1016/S0166-2236(98)01349-6 10322493

[B4] Belenky M. A. Pickard G. E. (2001). Subcellular distribution of 5-HT(1B) and 5-HT(7) receptors in the mouse suprachiasmatic nucleus. J. Comp. Neurol. 432, 371–388. 10.1002/cne.1109 11246214

[B5] Bonaventure P. Nepomuceno D. Hein L. Sutcliffe J. G. Lovenberg T. Hedlund P. B. (2004). Radioligand binding analysis of knockout mice reveals 5-hydroxytryptamine(7) receptor distribution and uncovers 8-hydroxy-2-(di-n-propylamino)tetralin interaction with alpha(2) adrenergic receptors. Neuroscience 124, 901–911. 10.1016/j.neuroscience.2004.01.014 15026130

[B6] Canese R. Adriani W. Marco E. M. De Pasquale F. Lorenzini P. De Luca N.. (2009). Peculiar response to methylphenidate in adolescent compared to adult rats: a phMRI study. Psychopharmacology (Berl.) 203, 143–153. 10.1007/s00213-008-1379-1 18998111

[B7] Canese R. Marco E. M. De Pasquale F. Podo F. Laviola G. Adriani W. (2011). Differential response to specific 5-Ht(7) versus whole-serotonergic drugs in rat forebrains: a phMRI study. Neuroimage 58, 885–894. 10.1016/j.neuroimage.2011.06.089 21763429

[B8] Canese R. Zoratto F. Altabella L. Porcari P. Mercurio L. De Pasquale F.. (2014). Persistent modification of forebrain networks and metabolism in rats following adolescent exposure to a 5-HT7 receptor agonist. Psychopharmacology (Berl.). [Epub ahead of print]. 10.1007/s00213-014-3639-6 24923983

[B9] Duncan M. J. Short J. Wheeler D. L. (1999). Comparison of the effects of aging on 5-HT7 and 5-HT1A receptors in discrete regions of the circadian timing system in hamsters. Brain Res. 829, 39–45. 10.1016/S0006-8993(99)01311-6 10350528

[B10] Ehlen J. C. Grossman G. H. Glass J. D. (2001). *In vivo* resetting of the hamster circadian clock by 5-HT7 receptors in the suprachiasmatic nucleus. J. Neurosci. 21, 5351–5357. 11438611 10.1523/JNEUROSCI.21-14-05351.2001 PMC6762851

[B11] Fellin T. (2009). Communication between neurons and astrocytes: relevance to the modulation of synaptic and network activity. J. Neurochem. 108, 533–544. 10.1111/j.1471-4159.2008.05830.x 19187090

[B12] Ferrari L. Turrini G. Crestan V. Bertani S. Cristofori P. Bifone A.. (2012). A robust experimental protocol for pharmacological fMRI in rats and mice. J. Neurosci. Methods 204, 9–18. 10.1016/j.jneumeth.2011.10.020 22068031

[B13] Fukuda M. Vazquez A. L. Zong X. Kim S. G. (2013). Effects of the alpha(2)-adrenergic receptor agonist dexmedetomidine on neural, vascular and BOLD fMRI responses in the somatosensory cortex. Eur. J. Neurosci. 37, 80–95. 10.1111/ejn.12024 23106361 PMC3538949

[B14] Glass J. D. Chen L. (1999). Serotonergic modulation of astrocytic activity in the hamster suprachiasmatic nucleus. Neuroscience 94, 1253–1259. 10.1016/S0306-4522(99)00369-3 10625065

[B15] Goshtasby A. (1988). Image registration by local approximation methods. Image Vis. Comput. 6, 255–261 10.1016/0262-8856(88)90016-9

[B16] Gozzi A. Schwarz A. Crestan V. Bifone A. (2008). Drug-anaesthetic interaction in phMRI: the case of the psychotomimetic agent phencyclidine. Magn. Reson. Imaging 26, 999–1006. 10.1016/j.mri.2008.01.012 18486387

[B17] Hagan J. J. Price G. W. Jeffrey P. Deeks N. J. Stean T. Piper D.. (2000). Characterization of SB-269970-A, a selective 5-HT(7) receptor antagonist. Br. J. Pharmacol. 130, 539–548. 10.1038/sj.bjp.0703357 10821781 PMC1572114

[B18] Hedlund P. B. Leopoldo M. Caccia S. Sarkisyan G. Fracasso C. Martelli G.. (2010). LP-211 is a brain penetrant selective agonist for the serotonin 5-HT(7) receptor. Neurosci. Lett. 481, 12–16. 10.1016/j.neulet.2010.06.036 20600619 PMC2910240

[B19] Hedlund P. B. Sutcliffe J. G. (2004). Functional, molecular and pharmacological advances in 5-HT7 receptor research. Trends Pharmacol. Sci. 25, 481–486. 10.1016/j.tips.2004.07.002 15559250

[B20] Hirst W. D. Price G. W. Rattray M. Wilkin G. P. (1997). Identification of 5-hydroxytryptamine receptors positively coupled to adenylyl cyclase in rat cultured astrocytes. Br. J. Pharmacol. 120, 509–515. 10.1038/sj.bjp.0700921 9031757 PMC1564482

[B21] Hodkinson D. J. De Groote C. McKie S. Deakin J. F. Williams S. R. (2012). Differential effects of anaesthesia on the phMRI response to acute ketamine challenge. Br. J. Med. Med. Res. 2, 373–385. 10.9734/BJMMR/2012/1412 22737655 PMC3378209

[B22] Kobe F. Guseva D. Jensen T. P. Wirth A. Renner U. Hess D.. (2012). 5-HT7R/G12 signaling regulates neuronal morphology and function in an age-dependent manner. J. Neurosci. 32, 2915–2930. 10.1523/JNEUROSCI.2765-11.2012 22378867 PMC3369253

[B23] Kocsis P. Gajari D. Deli L. Gocze K. Z. Pozsgay Z. Tihanyi K. (2013). Effect of tolperisone on the resting brain and on evoked responses, an phMRI BOLD study. Brain Res. Bull. 99, 34–40. 10.1016/j.brainresbull.2013.09.008 24099980

[B24] Leopoldo M. Lacivita E. Berardi F. Perrone R. Hedlund P. B. (2011). Serotonin 5-HT7 receptor agents: structure-activity relationships and potential therapeutic applications in central nervous system disorders. Pharmacol. Ther. 129, 120–148. 10.1016/j.pharmthera.2010.08.013 20923682 PMC3031120

[B25] Leopoldo M. Lacivita E. De Giorgio P. Fracasso C. Guzzetti S. Caccia S.. (2008). Structural modifications of N-(1,2,3,4-tetrahydronaphthalen-1-yl)-4-aryl-1-piperazinehexanamides: influence on lipophilicity and 5-HT7 receptor activity. Part III. J. Med. Chem. 51, 5813–5822. 10.1021/jm800615e 18800769

[B26] Liu X. Li R. Yang Z. Hudetz A. G. Li S. J. (2012). Differential effect of isoflurane, medetomidine, and urethane on BOLD responses to acute levo-tetrahydropalmatine in the rat. Magn. Reson. Med. 68, 552–559. 10.1002/mrm.23243 22213080 PMC3376170

[B27] Lukasik V. M. Gillies R. J. (2003). Animal anaesthesia for *in vivo* magnetic resonance. NMR Biomed. 16, 459–467. 10.1002/nbm.836 14696002

[B28] Martinez-Garcia E. Leopoldo M. Lacivita E. Terron J. A. (2014). Increase of capsaicin-induced trigeminal Fos-like immunoreactivity by 5-HT(7) receptors. Headache 51, 1511–1519. 10.1111/j.1526-4610.2011.02011.x 22082421 PMC3836505

[B29] Meneses A. (2004). Effects of the 5-HT7 receptor antagonists SB-269970 and DR 4004 in autoshaping Pavlovian/instrumental learning task. Behav. Brain Res. 155, 275–282. 10.1016/j.bbr.2004.04.026 15364487

[B30] Meneses A. Perez-Garcia G. Liy-Salmeron G. Ponce-López T. Lacivita E. Leopoldo M. (2014). 5-HT receptor activation: procognitive and antiamnesic effects. Psychopharmacology (Berl.). [Epub ahead of print]. 10.1007/s00213-014-3693-0 25074446

[B31] Meuser T. Pietruck C. Gabriel A. Xie G. X. Lim K. J. Pierce Palmer P. (2002). 5-HT7 receptors are involved in mediating 5-HT-induced activation of rat primary afferent neurons. Life Sci. 71, 2279–2289. 10.1016/S0024-3205(02)02011-8 12215375

[B32] Mnie-Filali O. Faure C. Lambas-Senas L. El Mansari M. Belblidia H. Gondard E.. (2011). Pharmacological blockade of 5-HT7 receptors as a putative fast acting antidepressant strategy. Neuropsychopharmacology 36, 1275–1288. 10.1038/npp.2011.13 21326194 PMC3079839

[B33] Mullins U. L. Gianutsos G. Eison A. S. (1999). Effects of antidepressants on 5-HT7 receptor regulation in the rat hypothalamus. Neuropsychopharmacology 21, 352–367. 10.1016/S0893-133X(99)00041-X 10457532

[B34] Neumaiera J. F. Sextona T. J. Yrachetab J. Diazb A. M. Brownfield M. (2001). Localization of 5-HT7 receptors in rat brain by immunocytochemistry, *in situ* hybridization, and agonist stimulated cFos expression. J. Chem. Neuroanat. 21, 63–73. 10.1016/S0891-0618(00)00092-2 11173221

[B35] Nilsson T. Longmore J. Shaw D. Pantev E. Bard J. A. Branchek T.. (1999). Characterisation of 5-HT receptors in human coronary arteries by molecular and pharmacological techniques. Eur. J. Pharmacol. 372, 49–56. 10.1016/S0014-2999(99)00114-4 10374714

[B36] Paxinos G. Watson C. (1998). The Rat Brain in Streotaxic Coordinates. San Diego, CA: Academic Press Inc.

[B37] Pierce P. A. Xie G. X. Meuser T. Peroutka S. J. (1997). 5-Hydroxytryptamine receptor subtype messenger RNAs in human dorsal root ganglia: a polymerase chain reaction study. Neuroscience 81, 813–819. 10.1016/S0306-4522(97)00235-2 9316030

[B38] Rojas P. S. Neira D. Munoz M. Lavandero S. Fiedler J. L. (2014). Serotonin (5-HT) regulates neurite outgrowth through 5-HT1A and 5-HT7 receptors in cultured hippocampal neurons. J. Neurosci. Res. 92, 1000–1009. 10.1002/jnr.23390 24752854

[B39] Romano E. Ruocco L. A. Nativio P. Lacivita E. Ajmone-Cat M. A. Boatto G.. (2014). Modulatory effects following subchronic stimulation of brain 5-HT7-R system in mice and rats. Rev. Neurosci. 25, 383–400. 10.1515/revneuro-2014-0007 24598832

[B40] Ruocco L. A. Treno C. Gironi Carnevale U. A. Arra C. Boatto G. Nieddu M.. (2014a). Prepuberal stimulation of 5-HT7-R by LP-211 in a rat model of hyper-activity and attention-deficit: permanent effects on attention, brain amino acids and synaptic markers in the fronto-striatal interface. PLoS ONE 9:e83003. 10.1371/journal.pone.0083003 24709857 PMC3977819

[B41] Ruocco L. A. Treno C. Gironi Carnevale U. A. Arra C. Mattern C. Huston J. P.. (2014b). Prepuberal intranasal dopamine treatment in an animal model of ADHD ameliorates deficient spatial attention, working memory, amino acid transmitters and synaptic markers in prefrontal cortex, ventral and dorsal striatum. Amino Acids 46, 2105–2122. 10.1007/s00726-014-1753-8 24862315

[B42] Shimizu M. Nishida A. Zensho H. Yamawaki S. (1996). Chronic antidepressant exposure enhances 5-hydroxytryptamine7 receptor-mediated cyclic adenosine monophosphate accumulation in rat frontocortical astrocytes. J. Pharmacol. Exp. Ther. 279, 1551–1558. 8968382

[B43] Shirayama Y. Muneoka K. T. Takigawa M. Minabe Y. (2001). Adenosine A2A, 5-HT1A and 5-HT7 receptor in neonatally pregnenolone-treated rats. Neuroreport 12, 3773–3776. 10.1097/00001756-200112040-00034 11726792

[B44] Sicard K. Shen Q. Brevard M. E. Sullivan R. Ferris C. F. King J. A.. (2003). Regional cerebral blood flow and BOLD responses in conscious and anesthetized rats under basal and hypercapnic conditions: implications for functional MRI studies. J. Cereb. Blood Flow Metab. 23, 472–481. 10.1097/00004647-200304000-00011 12679724 PMC2989608

[B45] Sommers M. G. Van Egmond J. Booij L. H. Heerschap A. (2009). Isoflurane anesthesia is a valuable alternative for alpha-chloralose anesthesia in the forepaw stimulation model in rats. NMR Biomed. 22, 414–418. 10.1002/nbm.1351 19003937

[B46] Steward C. A. Marsden C. A. Prior M. J. Morris P. G. Shah Y. B. (2005). Methodological considerations in rat brain BOLD contrast pharmacological MRI. Psychopharmacology (Berl.) 180, 687–704. 10.1007/s00213-005-2213-7 15778890

[B47] Ullmer C. Schmuck K. Kalkman H. O. Lubbert H. (1995). Expression of serotonin receptor mRNAs in blood vessels. FEBS Lett. 370, 215–221. 10.1016/0014-5793(95)00828-W 7656980

[B48] Weber R. Ramos-Cabrer P. Wiedermann D. Van Camp N. Hoehn M. (2006). A fully noninvasive and robust experimental protocol for longitudinal fMRI studies in the rat. Neuroimage 29, 1303–1310. 10.1016/j.neuroimage.2005.08.028 16223588

[B49] Wesolowska A. Nikiforuk A. Stachowicz K. Tatarczynska E. (2006). Effect of the selective 5-HT7 receptor antagonist SB 269970 in animal models of anxiety and depression. Neuropharmacology 51, 578–586. 10.1016/j.neuropharm.2006.04.017 16828124

