# Supplementary figures and images for: Differential responses to acute administration of a new 5-HT7-R agonist as a function of adolescent pre-treatment: phMRI and immuno-histochemical study
Source: Front Behav Neurosci. 2014 Dec 16;8:427. doi: 10.3389/fnbeh.2014.00427 (PMC4267273; doi:10.3389/fnbeh.2014.00427)

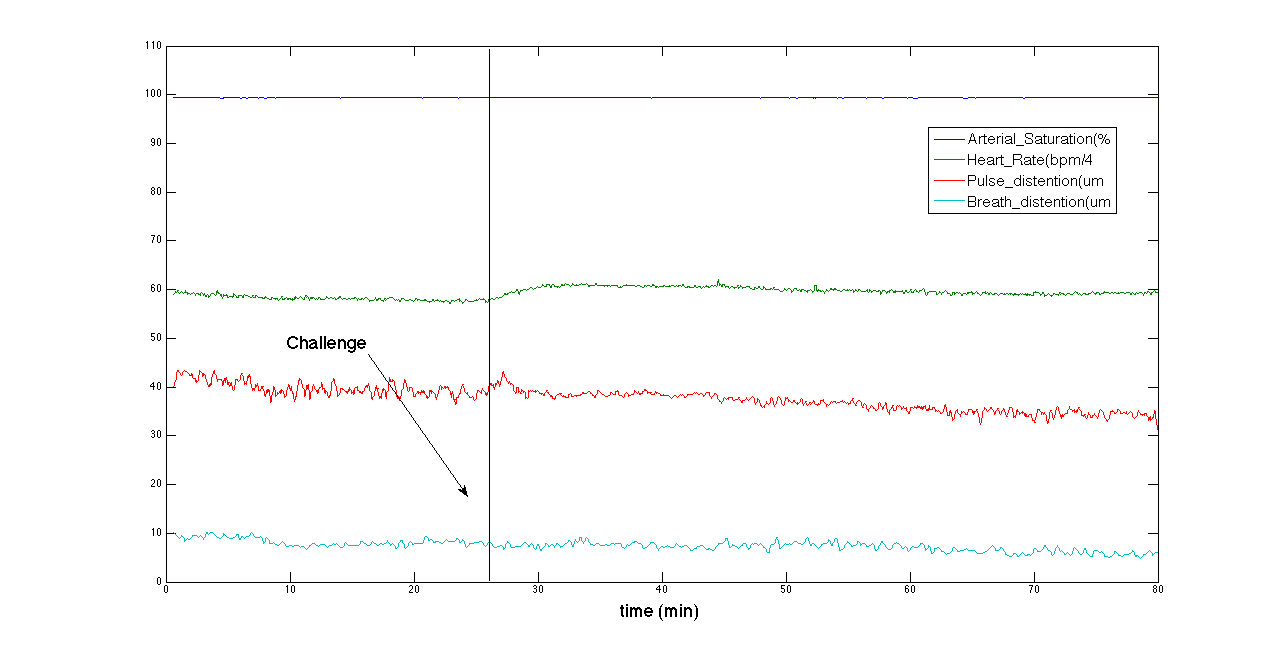

Supplement: Supplementary Figure 1 — An example of physiological time courses in a LP-211 challenged rat. After drug administration, there is only a transient increase of pulse distension and a slight and stable increase of heart rate. None of these time courses is coupled with the BOLD one, thus excluding any bias due to systemic effects. [file Image1.TIF]
